# Results of the re-introduction of the Griffon Vulture (*Gypsfulvus*) in Vrachanski Balkan Nature Park, Bulgaria – completion of the establishing phase 2010–2020

**DOI:** 10.3897/BDJ.11.e100834

**Published:** 2023-04-04

**Authors:** Georgi Stoyanov, Hristo Valeriev Peshev, Elena Kmetova–Biro, Emilian Stoynov, Ivelin Ivanov, Nadya Vangelova, Zlatka Nikolova, Emanuil Mitrevichin, Atanas Grozdanov

**Affiliations:** 1 Birds of Prey Protection Society, 23 Golyam Bratan Str., www.bpps.org, Sofia, Bulgaria Birds of Prey Protection Society, 23 Golyam Bratan Str., www.bpps.org Sofia Bulgaria; 2 Fund for Wild Flora & Fauna, 49 Ivan Mikhaylov Str., office 327, P.O.Box 78, www.fwff.org, pirin@fwff.org, Blagoevgrad, Bulgaria Fund for Wild Flora & Fauna, 49 Ivan Mikhaylov Str., office 327, P.O.Box 78, www.fwff.org, pirin@fwff.org Blagoevgrad Bulgaria; 3 Green Balkans – www.greenbalkans.org, Stara Zagora, 9 Stara Planina Str., Bulgaria Green Balkans – www.greenbalkans.org Stara Zagora, 9 Stara Planina Str. Bulgaria; 4 Central European University, Department of Environmental Sciences and Policy, Vienna, Austria Central European University, Department of Environmental Sciences and Policy Vienna Austria; 5 South-West University „Neofit Rilski“, Faculty of Mathematics and Natural Sciences, Department of Geography, Ecology and Environmental Protection, Blagoevgrad, Bulgaria South-West University „Neofit Rilski“, Faculty of Mathematics and Natural Sciences, Department of Geography, Ecology and Environmental Protection Blagoevgrad Bulgaria; 6 Department of Zoology and anthropology, Faculty of Biology, Sofia University “St. Kliment Ohridski”, 8 Dragan Tsankov Blvd, zootribe@gmail.com, Sofia, Bulgaria Department of Zoology and anthropology, Faculty of Biology, Sofia University “St. Kliment Ohridski”, 8 Dragan Tsankov Blvd, zootribe@gmail.com Sofia Bulgaria

**Keywords:** re-establishment, acclimatisation, releases, feeding site, home range, Vrachanski Balkan SPA, raptor, conservation

## Abstract

The current study analyses and presents the results of the ten-year establishment phase of the Griffon Vulture (*Gypsfulvus*) local re-introduction in Vrachanski Balkan Nature Park, north-western Bulgaria. Between 2010 and 2020, 61 rehabilitated and captive-bred Griffon Vultures from Spain, France and several European zoos were released from an acclimatisation aviary. The first successful breeding in the wild was reported in 2015. Thus, the species has been restored as a nesting species in the area after more than 60 years of absence. In 2020, the local population accounted for some 55–70 individuals, consisting of about 20–23 breeding pairs in three-five separate colonies and two frequently-used roosting sites. Forty-two chicks fledged from 2010 to 2020, at an average breeding success of 0.46 chicks/territorial pair and productivity of 0.62 fledglings/breeding pair. The mortality rate is calculated at 0.34; an additional 0.07 of the released individuals have never been seen or found. The local nucleus of the Griffon Vulture now covers a territory of 1,478.58 km², calculated as a 95% home range, while the 50% core area is 9.07 ± 5.73 km^2^ (range 2.12–22.89 km^2^). With these results, we consider the establishment phase of the re-introduction of the species in Vrachanski Balkan Nature Park as completed.

## Introduction

The Griffon Vulture *Gypsfulvus* (Hablizl, 1783) was reported as numerous and widespread throughout Bulgaria up to the 1940s (Patev 1950, Demerdzhiev et al. 2007). Starting to recover from the lowest point of 1–4 pairs in the late 1970s, the last remaining autochthonous colonies of Griffon Vultures in the Eastern Rhodopes gradually increased in numbers reaching over 100 pairs in 2020, yet only remaining in the Arda River Valley (Demerdzhiev et al. 2014, Dobrev and Stoychev 2014, Dobrev et al. 2021), showing no ability to naturally expand to other parts of Bulgaria (Stoynov et al. 2018b).

The Griffon Vulture is assumed with high reliability to have nested at a total of seven sites in the area of Vrachanski Balkan Nature Park (hereafter VBNP), north-western Bulgaria until about the 1950s (Stoyanov 2010a, Stoyanov 2010b). After that, the species declined and became locally extinct as a breeder around 1970, mainly due to poison baits used to exterminate mammalian predators (Baumgart 1974, Demerdzhiev et al. 2007). Furthermore, there was a long period when the Griffon Vulture was considered absent from the area (Patev 1950, Boev and Mitchev 1981, Yankov and Profirov 1991, Demerdzhiev et al. 2007, Demerdzhiev et al. 2014).

Due to the unique role of vultures in ecosystems (naturally utilising livestock and wild ungulates carcasses) and considering the current state of the environment and threats, an international initiative called Action Plan for the Recovery and Conservation of Vultures on the Balkan Peninsula (BVAP) (Tewes et al. 2004) was developed. One of the plan's aims was securing the long-term survival of the Griffon Vulture population in Bulgaria, increasing its numbers and expanding its breeding range through strategic re-establishment of colonies in former breeding sites, while also serving as proxy species for restoration of the rarer Cinereous (*Aegypiusmonachus*) and Bearded (*Gypaetusbarbatus*) Vultures.

One of the areas assessed as potentially suitable for local re-introduction was VBNP, based on geographic features (extensive inland cliffs), historical presence and re-introduction management capacity (e.g. local NGO activists and a Nature Park administration). Extensive preparatory work, field inventories and test feedings resulted in the compilation of a detailed Viability Study for the re-introduction of the Griffon Vulture in VBNP (Stoyanov et al. 2006). The practical re-introduction started in 2010 with the release of the first group of Griffon Vultures imported from Spain. The local initiative was a part of a large-scale national process to restore former colonies of the species, with simultaneous releases in the Eastern Balkan Mountains (Sinite Kamani Nature Park and Kotlesnka Planina SPA) (Kmetova-Biro et al. 2021), Central Balkan National Park and Kresna SPA (Stoynov et al. 2018b).

The birds released between 2010 and 2020 were either wild-born individuals originating from rehabilitation centres in Spain or France or captive-bred vultures originating from various European zoos. Different age groups were used: juvenile, adult, but primarily immature individuals. The vultures are being accommodated in a provisionally built adaptation aviary and soft released in line with the methodology presented by Terrasse and Choisy (2007), developed and applied in Massif Central, France, in the 1980s (Terrasse 2006).

The first breeding behaviour observed in the wild was reported in 2013 and the first successfully hatched chick was confirmed in 2015, becoming the first successfully fledged wild-born Griffon Vulture amongst all active release sites in Bulgaria. By then, 44 birds had already been released in VBNP, mostly juveniles (Stoyanov et al. 2016).

The tenth year from the start of the Griffon Vulture releases in VBNP is an important milestone. Furthermore, the establishment phase (in accordance with Armstrong and Seddon (2008)) of the local re-introduction of the species has been completed. The current study provides an overview of the success of this local re-introduction initiative, describing and analysing the number of vultures released, their survival, emigration, breeding attempts, breeding success, causes of mortality, demographic parameters and the home range of the re-established breeding nucleus in VBNP at the end of 2020.

## Material and methods

**Figure 1.**
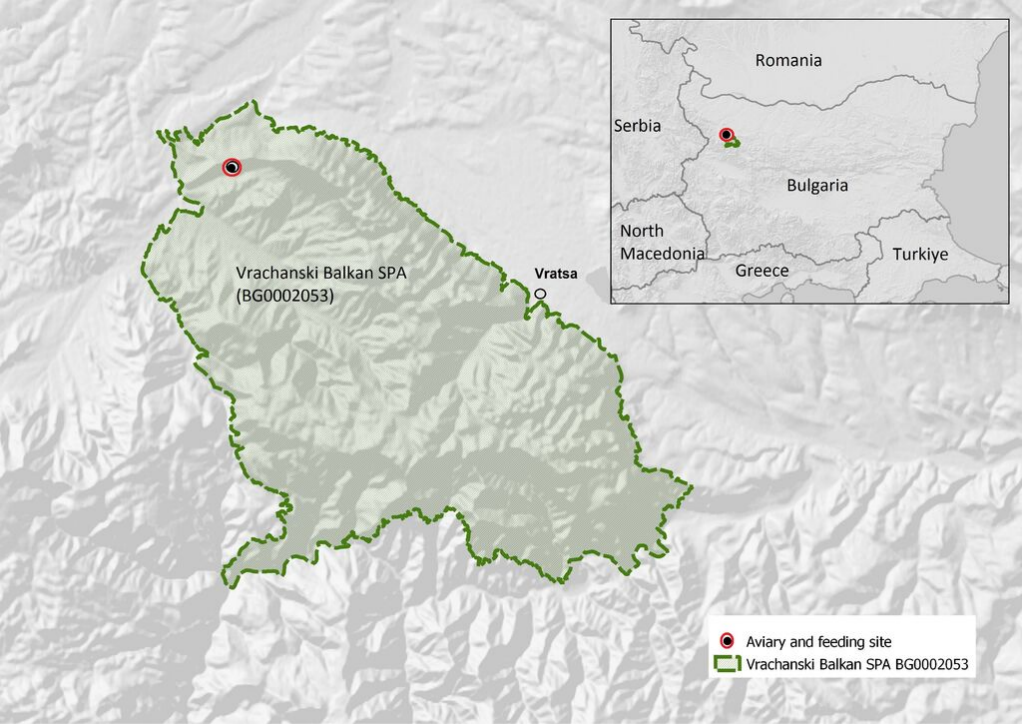
A map of the Vrachanski Balkan SPA and the Griffon Vulture release and project feeding site.

For the current analysis, the "release area" is referred to as the Vrachanski Balkan SPA (BG0002053) (see Fig. 1), entirely comprising the territory of Vrachanski Balkan Nature Park (UTM, FN99) but covering a larger total area of about 2,827 km^2^ (30 km radius with the centre being the village of Zgorigrad in VBNP) following the expected use of the territory of the Griffon Vulture (Terrasse and Choisy 2007).

From October 2010 to the end of 2020, 52 Griffon Vultures were released in VBNP. All of the birds were marked with standard metal ornithological rings, PVC rings and wing tags with matching inscriptions to enable and ease individual identification and monitoring. The numbers of released individuals by year are shown in Table 1.

A fenced feeding site was set up in front of the acclimatisation aviary to ensure the fixing of the birds to the release site and assist the monitoring (Terrasse and Choisy 2007). Since the start of the Griffon Vulture releases in 2010, food has been provided at least twice a week, comprising livestock carcasses (sheep, cattle, horses and pigs) and slaughter offal. A camera trap was set at the feeding site, being checked every 3–5 days. The total number of the vultures seen in the photographs and all tags identified were filled in a database. In addition, the feeding site was monitored once a week by a spotting scope (20-60X) from a distance of 600 m to count the total number of the vultures present.

All potentially suitable cliffs in the area of release and the adjacent areas were surveyed for nests occupied between 2010 and 2020. The monitoring was implemented each year from January to August, visiting each cliff a minimum of once a week. Observations were carried out in good weather and visibility, at a distance of 300 to 1300 m from the particular cliff to avoid disturbance, using spotting scopes. Following Demerdzhiev et al. (2014), a cliff was considered a Griffon Vulture colony when at least two pairs occupied it at a distance of at least one kilometre from the neighbouring occupied cliff. The breeding birds (almost all successfully identified through their markings – wing tags and colour rings), nest location and occupation time and activity (nest building, mating, laying, chick rearing) were recorded for every nest. Other parameters recorded (in line with Henriquet et al. (2012) for comparison) were:

1. the number of occupied nests (all nests occupied by breeding and non-breeding pairs),
2. the number of breeding pairs (pairs that were observed incubating),
3. breeding success (fledged juveniles per incubating pairs),
4. productivity (fledged juveniles per occupied nest),
5. hatching success (hatchlings per incubating pair).

All of the Griffon Vulture nests discovered so far have been located on cliffs. Nest site preferences are, thus, only considered in terms of altitude and geographic orientation, as well as the distance to the feeding site and nearby villages.

In line with Demerdzhiev et al. (2014), the criteria for counting non-breeding pairs (pairs that did not lay eggs) was an observation in which both birds from the pair were attached to a particular niche (ledge) of a potential breeding cliff, where the nest may or may not have been present; and engage in at least two of the following behaviour types: courtship flights, mutual preening, copulation, nest building and defence of the immediate vicinity of the chosen nest site from conspecifics. A juvenile was considered fledged when it reached an age of at least 125 days (Cramp and Simmons 1980) and, for the marked ones, when seen perched out of the nest or flying in the area.

Breeding success, fledging success, survival rate, mortality factors and demographic parameters were calculated, based on annual averages and percentages. In addition, the home range of the Griffon Vulture in VBNP was calculated, based on data provided by 17 individuals tagged with GPS-GSM transmitters in 2017–2021. OrniTrack - transmitters (produced by Ornitela, Vilnius, Lithuania) were attached to the patagium with the vinyl wing tag - OTP-33 or through leg-loop OT-30 and OT-50 to the lower back of the birds. The devices weighed from 33 to 50 g which is ca. 1% of the body mass (< 3% is recommended for flying birds) and a vulnerable attaching element was deliberately used to ensure that the device would fall after a couple of years. Each transmitter placement took less than ten minutes and was mounted while the bird's head was covered to ensure minimal stress.

**Home range estimations**

The home range of the vultures released in the VBNP was calculated, based on 4,429 tracking days of 17 tagged vultures, comprising a dataset of over 224,529 GPS fixes. Here, we analyse tracking days and GPS fixes. The home range of each vulture was calculated using the dynamic Brownian bridge movement model (dBBMM) (Kranstauber et al. 2012). Data were statistically processed using the open-source R software environment for statistical computing and graphics Version 4.0.3 (R Core Team 2020) and the packages "adehabitatHR" (v.0.4.18; Calenge (2006), Calenge (2019)) and "move" (v.4.0.6; Kranstauber (2020)). A 95% home range was defined as the general individual home range and 50% home range was defined as the core area.

The GPS tracking data were also used to analyse the feeding events and compare the relative use of food provided at the supplementary feeding site or randomly found in nature. The transmitters were set to take GPS fixes every 10 min. Feeding at the feeding site was considered the landing of the tracked vulture on-site and staying for over 20 min when the carcass was available (following disposal or observed presence). Feeding out of the feeding site was considered when a tracked individual landed on a carcass in the field (confirmed through direct inspection) or when more than one tracked bird was landing in an open area out of the traditional roosting and watering sites, spending more than 20 min there.

## Results and discussion

### Survival, fate, emigration and immigration

In December 2020, 0.60 (n = 31) of all released vultures in VBNP survived, while 0.87 of these birds (n = 27) settled in the area. Immigration of birds released in Central Balkan National Park (Stoynov et al. 2018b) boosted the local population of VBNP (see Table 2).

**Figure 2.**
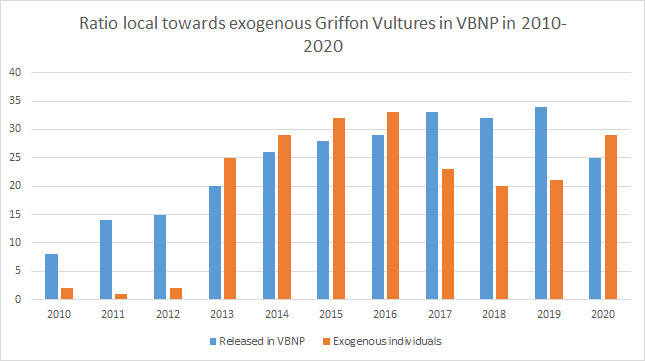
Dynamics of the number and ratio of the local to exogenous Griffon Vultures present in VBNP in 2010–2020.

The aggregate number of individually identified Griffon Vultures observed in the VBNP per year has gradually increased from 6–13 individuals in 2010 to an average of 41 in 2020. At the re-introduction programme beginning in 2010, 0.80 of the individuals observed were released in the area and gradually decreased to 0.44 in 2013, reaching values between 0.45 and 0.62 in the period 2016–2020 (see Fig. 2). Between 2016 and 2020 the highest concentration of birds was reported from May to August (48 individuals on average, max. = 62), while the lowest numbers were recorded from January to March (33 individuals on average, max. = 44) (see Table 3). These numbers reflect the growing importance of VBNP for the vultures released locally and as a core area offering suitable conditions, attracting and retaining many exogenous birds. The maximum aggregate number of 62 different vultures visiting the site per year was reported in 2019 before a large-scale poisoning accident in September of the same year. In addition to the birds released in the area, vultures originating from the three other release sites: Central Balkan, the Eastern Balkan Mountains, Kresna SPA, as well as the natural populations from the Eastern Rhodopi Mountains (Bulgaria), Serbia, Croatia and Israel, have been identified on site. It should be noted that single individuals tagged in Italy, Greece and North Macedonia were reported for the first time on-site in 2020. The gradually increasing number of vultures that come from different sites to the VBNP area to forage and roost is further evidence of the significance and critical location of the VBNP area.

### Mortality rate and reasons

Twenty-one vultures released in the VBNP were confirmed dead in 2010–2020 (see Table 4), which accounts for 0.40 confirmed mortality. At the same time, it should be noted that there are no further observations and data for the survival of four of the vultures released (0.07). A total of 0.62 (n = 13) from all mortality cases occurred within the release area. Poisoning is estimated to have caused more than half (0.52) of the reported mortality cases, mainly due to the single large-scale poisoning incident from September 2019 near the village of Bov, Svoge Municipality. The case was quickly located with the help of the GPS transmitters carried by two of the poisoned vultures. The immediate on-site inspection found two dead Griffon Vultures and a Golden Eagle (*Aquilachrysaetus*) around a calf carcass. Two more dead Griffon Vultures were found a day later. More reports of vulture corpses found in the area in the following months estimated the total loss at about 20 vultures (Peshev et al. 2022). The poisoning is the most intense threat to vultures globally (Botha et al. 2017) and is hard to control in the Balkans because of the severe man-predators conflict (Parvanov et al. 2017), which is hard to control or mitigate, keeping in mind its spatio-temporal dynamics (Stoynov et al. 2018a). However, this threat is recently well under supervision by the conservation community with the application of intensive monitoring of vultures with GPS transmitters (Stoynov et al. 2019) and a broad spectrum of legal action by the Ministry of Environment and Waters in Bulgaria by adopting and applying a National Strategy Against the Use of Poison. The success of intensive GPS tracking and fast reaction in the field is especially relevant to large, infrequent poisoning episodes, such as the ones from Kresna Gorge in 2017 (Peshev et al. 2019) and Vrachanski Balkan in 2019 (Peshev et al. 2022). However, Tsiakiris et al. 2021 state that frequent minor poisoning episodes could harm marginal vulture colonies and populations much more. Thus, a holistic and wide-spectrum fight against poison use should be ongoing. Another significant mortality factor in the area is electrocution, which was the cause of death in 0.24 of the reported cases. The rest of the causes remain unidentified or random, such as emaciation (n = 3), road kill (n = 1) and predation (n = 1). Sarrazin et al. 1996 report 0.27 (n = 59) mortality or removal of the Griffon Vultures released between 1981 and 1991 due to emaciation or injuries, which is lower than the observed mortality in the VBNP.

### Breeding performance – number of pairs, colonies

**Figure 3.**
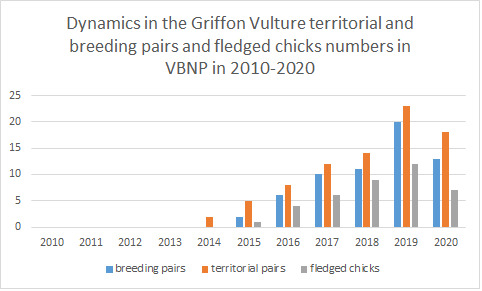
Dynamics in the Griffon Vulture territorial and breeding pairs and fledged chick numbers in VBNP in 2010–2020.

The first breeding behaviour of Griffon Vultures in the area was observed in 2013. Two years later, in 2015, five territorial pairs were confirmed and the very first chick successfully fledged in the wild (Stoyanov et al. 2016). Since then, in 2015–2020, 42 chicks have successfully fledged in the wild (see Table 5). The number of territorial pairs gradually increased to 23 in 2019. They produced 12 fledglings, but then dropped to 18 territorial pairs in 2020 following the large-scale poisoning accident in September 2019. (see Fig. 3). Unfortunately, in 2020, only 13 pairs bred and produced a total of seven successful fledglings. The average breeding success of the newly-restored VBNP population accounts for 0.46 fledglings/territorial pair and fledging success of 0.62 fledglings/breeding pair between 2015 and 2020. These numbers are even better than the productivity of 0.57 fledglings/breeding pair reported for an autochthonous small Griffon Vulture colony in north-western Spain (Olea et al. 1999), yet still lower than the mean breeding success of the natural population of Griffons in Bulgaria, reported at 0.77 ± 0.14 and average productivity of 0.71 ± 0.16 for the period 1987–2011 (Demerdzhiev et al. 2014). At the same time, Sarrazin et al. (1996) report nesting success of 0.42 fledglings/breeding pair for birds released when older than three years old and 0.82 for younger vultures released and wild-born birds within a re-introduction programme in Grand Causses, southern France, in 1982–1992.

Three to five distinct Griffon Vulture colonies are identified in VBNP – Kotlya, Medkovski Dol, Manastirski Dol, Vratsata and Iskar Gorge (above small hydropower plant "Svrazhen"). The latter has been occupied since 2020. Out of Iskar Gorge and Kotlya (the main colony), the other three are used irregularly, from one to three pairs a year each.

Importantly, locally released Griffon Vultures were amongst the main breeders confirmed in VBNP along with birds released in other local re-introduction sites – the Eastern Balkan Mountains (UTM, MH43 and MH65), Central Balkan National Park (UTM, LH32) and Kresna SPA (UTM, FM73) (Stoynov et al. 2018b). Non-tagged exogenous and locally-fledged individuals were also amongst the breeders.

### Number of the local population, source/sink balance

The Griffon Vultures, constantly present in VBNP in December 2020, accounted for about 55–70 individuals. Slightly less than half (0.46) of this number consists of locally-released birds, all ringed and tagged, allowing for individual identification and close monitoring. The area was also regularly visited by roaming or migrating immigrants from the autochthonous populations in Serbia and Croatia on the way to their summer or wintering grounds (see Table 6). The source/sink balance (in accordance with Pulliam (1988)) of the newly-established population nucleus of the Griffon Vulture in VBNP was assessed, extracting the total number of exogenous birds lost on site (n = 8) from the total number of successfully fledged Griffon Vultures in the area (n = 33) (see Table 6). The results are significantly impacted by a single misfortunate poisoning accident, which happened in 2019, yet the number of locally-produced individuals considerably exceeds the number of locally-lost exogenous birds between 2010 and 2020 (see Table 6). The total balance for the 10-year-period accounts for +21 individuals, which means that VBNP can be considered as one of the five Griffon Vulture population sources amongst all seven general species areas found in the mainland Balkans (Peshev et al. 2021).

### Home range estimation

The 95% home range of 17 Griffon Vultures tracked in the Vrachanski Balkan Nature Park is estimated at: 459.24 ± 230.78 km^2^ (range 146.61–1083.83 km^2^); 50% core area: 9.07 ± 5.73 km^2^ (range 2.12–22.89 km^2^) (see Table 7). This is significantly less than the estimation of 4,957 km^2^ published by Stoynov et al. (2018b). The discrepancy is, however, likely due to different calculation methods (KDE instead of dBBMM) as opposed to a genuine decrease of the utilised territory.

**Figure 4.**
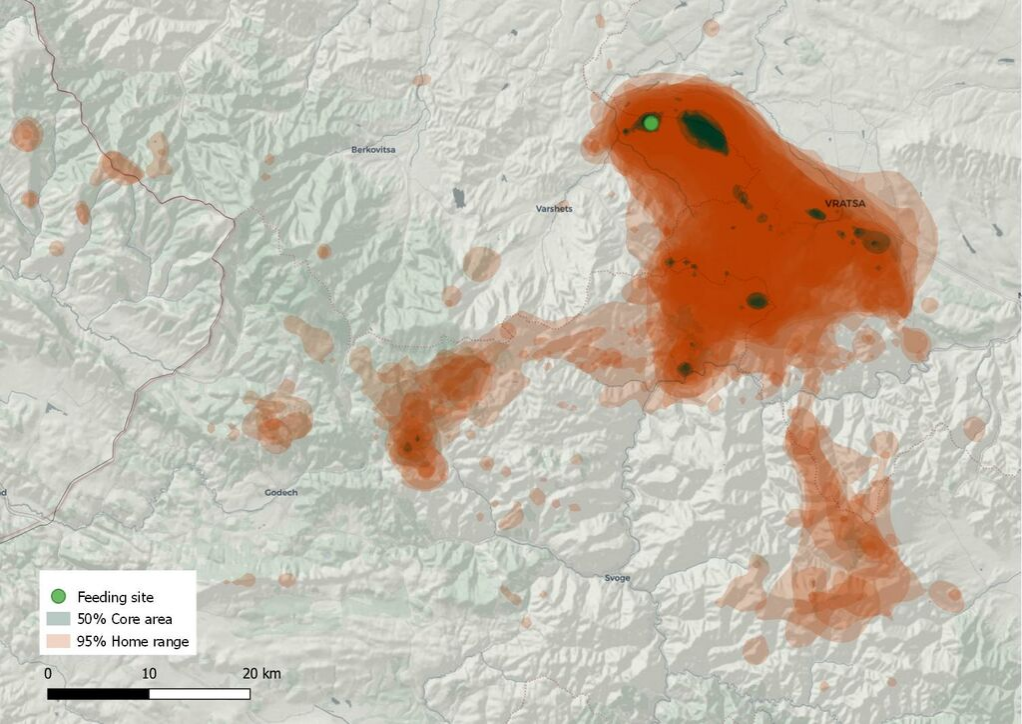
A map of the home range of the newly-established breeding nucleus of the Griffon Vulture in VBNP.

The total coverage of all vulture core areas, obtained by overlapping all acquired 50% polygons, was estimated at 42.02 km² and the 95% home range was similarly calculated at 1,478.58 km², which can be considered as the actual range of the Griffon Vulture in the region (see Fig. 4).

### Feeding on vulture restaurants and in the wild

The frequency and the amounts of food provided to the project feeding site near the Dolno Ozirovo Village in VBNP increased steadily from 2010 to 2016 – from 12 to more than 60 tonnes and then remained stable at about 140–190 feedings and total yearly provision of about 55–59 tonnes. Table 8 summarises the number of food provisions and the total quantity per year provided to the vulture feeding site in VBNP in 2010–2020.

The Table 9 summarises the Griffon Vulture presence at the feeding site – maximum number per month for 2010–2020.

**Figure 5.**
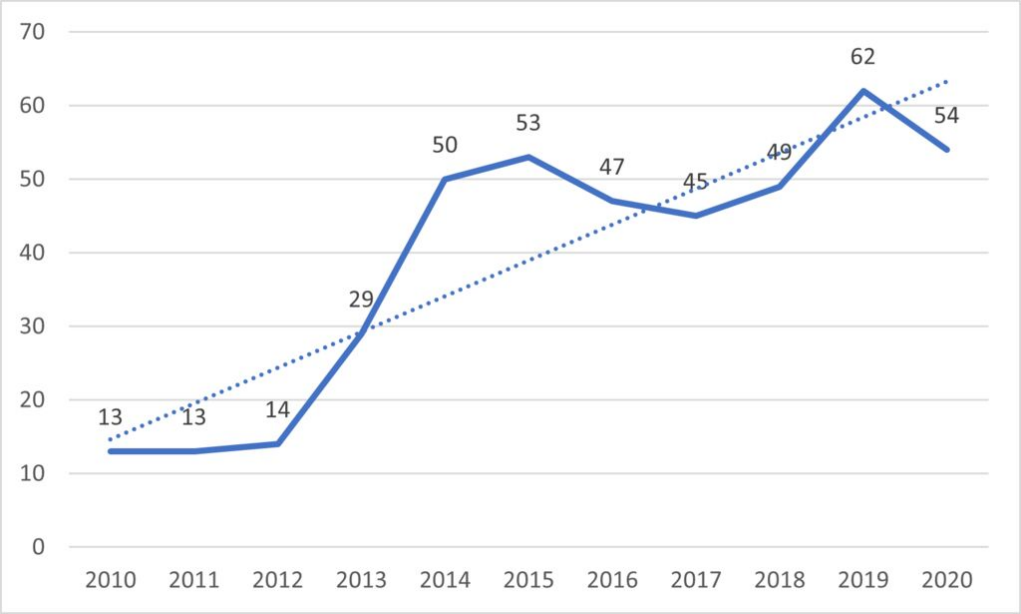
Maximum aggregate number of Griffon Vultures observed within a single month at the project feeding site in VBNP for the given year.

Fig. 5 visualises the general increase in the monthly numbers of Griffon Vultures individuals at the project feeding site.

The tracked Griffon Vultures in VBNP prefer foraging at the project feeding site (0.78 of the recorded feedings, n = 218), whereas 0.22 of the feedings (n = 62) were "wild". Within the territory of about 5,058 km^2^, there were feedings in 0.45 days of the time and 0.55 days were without feeding. A study by provides a detailed insight into the ratio of natural (wild) food use to vulture restaurants' use of the growing autochthonous Griffon Vulture population in the Eastern Rhodopes, Bulgaria. The study Arkumarev et al. (2021) suggests that 0.80 of the foraging events occur at wild food sources and the supplementary feeding sites only play a minor role. However, the dependence on wild feedings of the largest Griffon Vulture nucleus in the Balkan Peninsula, such as in the Gorge of Uvats and the adjacent regions in Serbia (Dobrev et al. 2021) in the winter period, is also scarce and they rely very much on the vulture restaurants (Marinković et al. 2020). In these regions, the feeding site usage by local Griffon Vultures is also about 0.80 (Stoynov 2019). The similarity in the vulture restaurants' dependence on the colonies in the Uvats Gorge and VBNP is related to the shared environmental features compared to the Eastern Rhodopes, where the climate is with a Meditteranean impact and the suitable foraging habitat and vulture colonies are distributed throughout a larger territory (e.g. also the Greek part of the mountain).

The obvious increase in free-ranging horses (mainly) and cattle in 2016–2020 in VBNP and adjacent territories – south-west above the town of Botevgrad, Ponor SPA – near the villages of Zimevitsa, Dobravitsa, Breze and Brakiovtsi, as well as the areas north of the town of Godech, is the reason the Griffon Vultures often visit and feed in these areas. Griffon Vultures also feed on hunting grounds, for example, the Private Game Breeding Area "Ledenika" in VBNP, where carcasses are provided to attract terrestrial predators. Larger and more frequently supplied carcasses are found in "Streshersky polyani" – south of Stresher peak; the locality "Darzhavnata polyana" – south of Sokolo peak and the locality "Dudil" near Buk peak. Additionally, in 2019, the Griffon Vultures fed on a carcass disposal site in the Ponor Mountains, south of Iskrets. At another place, "Babina Reka", north above the village of Breze in the Ponor Mountains, livestock carcasses are provided in front of a hide for wildlife photographers.

## Conclusions

The Griffon Vulture has been successfully re-introduced to Vrachanski Balkan Nature Park. The local nucleus now consists of 3-5 colonies, 55-70 individuals and 20-23 breeding pairs, which should be updated in the Standard Data Form of the Vrachanski Balkan SPA (BG0002053). Since the start of the re-introduction activities in 2010, the following criteria have been successfully met: 1) first successful breeding in the area; 2) a minimal number of 10 successfully fledged offspring a year on site; 3) first breeding of locally-fledged birds. We, therefore, consider the establishment phase (in accordance with Armstrong and Seddon (2008)) of this local re-introduction to be completed. The newly-established colonies in VBNP are forming a common refuge, acting as a source population with a positive balance of individuals produced and are now well integrated into the Balkan Griffon Vulture meta-population. Many birds originating from Serbia, Croatia, Eastern Rhodopes, Kresna and Eastern Balkan Mountains (Bulgaria) and others are using it for summering and wintering or during the migration and sojourn. The breeding success of the new colonies is around the average and higher for the Griffon Vulture in Europe. The newly-established population is, however, still vulnerable and threatened by accidental poisoning and should be actively supported with feeding site maintenance and adaptive management until a comprehensive modelling of the persistence phase (in accordance with Armstrong and Reynolds (2012)) is made and directions for future actions are provided in more detail. Thus, the food provision on the project feeding site (above the village of Dolno Ozirovo) in the northern parts of the VBNP must continue. The second vulture feeding site, built by the VBNP Directorate in the eastern part of the Park, is also necessary to start working. This would improve the feeding activities of the vultures and the extensive usage of the entire territory of the Park for foraging and breeding.

## Figures and Tables

**Table 1.** The number of released Griffon Vultures by year in Vrachanski Balkan Nature Park from 2010 to 2020.

Year	Number of Griffon Vultures released
2010	8
2011	7
2012	5
2013	8
2014	12
2015	3
2016	4
2017	0
2018	1
2019	2
2020	2
**Total**	**52**

**Table 2.** The number and origin of the Griffon Vultures identified in Vrachanski Balkan Nature Park for 2010–2020.

**Origin**	**2010**	**2011**	**2012**	**2013**	**2014**	**2015**	**2016**	**2017**	**2018**	**2019**	**2020**
Vrachanski Balkan Nature Park (locally released)	8	14	15	20	26	28	29	33	32	34	25
Central Balkan (re-introduced)	0	0	0	8	11	12	14	10	7	6	5
Eastern Balkan Mountains (re-introduced)	0	0	1	7	6	5	6	6	7	6	9
Kresna Gorge (re-introduced)	0	0	0	2	6	6	4	1	1	2	6
Eastern Rhodopes (autochthonous)	0	0	0	0	0	0	0	0	0	1	0
Serbia (autochthonous)	0	0	0	2	1	4	5	4	3	2	3
Croatia (autochthonous)	0	0	0	1	2	1	1	1	0	0	2
Israel (tagged there, but most probably of Balkan origin)	2	0	1	2	2	3	4	1	1	2	0
North Macedonia	0	0	0	0	0	0	0	0	0	0	1
Greece	0	0	0	0	0	0	0	0	0	0	1
Italy	0	0	0	0	0	0	0	0	0	0	1
Tagged exogenous unidentified	0	1	0	3	1	1	1	0	1	2	1

**Table 3.** The maximum number of Griffon Vultures observed in Vrachanski Balkan Nature Park by months and years (2010–2020). The numbers for each month represent the total number of individually identified vultures and the maximum number of non-tagged individuals observed for the given month. Therefore, the numbers do not reflect a group of vultures reported within a single observation.

Year	Month											
	Jan	Feb	Mar	Apr	May	Jun	Jul	Aug	Sept	Oct	Nov	Dec
2010								2	6	13	8	7
2011	6	7	8	8	9	9	9	10	12	12	11	13
2012	9	6	9	8	10	11	12	13	13	14	12	11
2013	12	12	7	24	21	28	26	23	29	19	20	19
2014	23	20	21	26	30	50	44	44	35	45	40	40
2015	40	40	40	37	43	40	50	48	53	47	43	46
2016	43	44	41	36	45	47	41	43	47	46	37	41
2017	37	27	37	25	44	41	43	45	40	41	40	39
2018	31	37	31	30	45	45	49	48	42	41	40	42
2019	25	29	29	41	62	52	54	61	31	36	28	21
2020	20	35	24	48	51	49	50	54	45	41	38	43

**Table 4.** The fate of the Griffon Vultures released in Vrachanski Balkan Nature Park for the period 2010–2020 – status at December 2020.

A. Number of dead individuals in the area of release VBNP (by reason)			B. Number of dead individuals outside the release area (by reason)			C. Breeds/sojourn anywhere out of the release area	D. Breeds/ sojourn in VBNP (the release area)	E. Unknown fate
electrocution	poison	other	electrocution	poison	other			
4	8	1	1	3	4			
13			8			3	24	
21						27		4
**52**								

**Table 5.** Breeding performance of the newly established Griffon Vulture local population in Vrachanski Balkan Nature Park for 2014–2020. The years with successful reproduction are given **in bold**.

Site	Year	# Colonies	# Territorial pairs (b)	# Breeding pairs (c)	# Fledglings (d)	Breeding success (d/b)	Fledging success (d/c)
Vrachanski Balkan NP UTM, FN99	2014	1	2	0	0	0	0
	**2015**	**1**	**5**	**2**	**1**	**0.20**	**0.50**
	**2016**	**2**	**8**	**6**	**4**	**0.50**	**0.67**
	**2017**	**2**	**12**	**10**	**6**	**0.50**	**0.60**
	**2018**	**2**	**14**	**11**	**9**	**0.64**	**0.81**
	**2019**	**2**	**23**	**20**	**12**	**0.52**	**0.60**
	**2020**	**3**	**18**	**14**	**10**	**0.55**	**0.71**

**Table 6.** Multi-annual dynamics of the number and mortality of the Griffon Vulture in VBNP with analysis of source/sink balance – the number of dead immigrants compared to the number of locally-fledged individuals.

Year	2010	2011	2012	2013	2014	2015	2016	2017	2018	2019	2020	**Total**
Max. number of Griffon Vultures observed	8	16	18	25	32	44	52	58	63	68	55	-
Number of dead individuals (of them immigrants)	0	1	2	1	0	0	(1)	1	0	15 (7)	0	**21 (8)**
Balance – locally-fledged to dead immigrants in the area	0	0	0	0	0	1	3	6	9	5	10	**34**

**Table 7.** Home range data for the individual birds tracked - days of tracking and number of GPS coordinates.

tag	50% Core area, km^2^	95% Home range, km^2^	days	points
1X	13.46	1,083.83	699	35,680
56	22.89	491.58	47	3,028
A4	10.58	619.28	678	27,252
A42020	5.42	249.35	20	1,173
C1-M	4.94	534.72	586	33,402
C5	10.76	676.08	147	11,842
E1	7.19	304.39	198	8,773
E3	5.10	319.21	93	5,692
F4	4.16	301.93	571	24,885
F6	5.96	310.02	577	34,778
P-B2F	3.81	146.61	42	2,159
V3	2.12	462.35	25	1,364
V6	12.98	338.31	13	709
XE	4.51	307.56	356	18,885
Y1	8.18	625.36	29	1,624
YE	13.02	320.55	52	2,943
Z7	18.95	715.82	296	10,340

**Table 8.** The quantity of food in kg provided to the project feeding site in VBNP per year for 2010–2020. * The figures for 2010 include only the last three months of the year – October, November and December – the period after the release of the first Griffon Vultures.

Food provisioned/year	2010*	2011	2012	2013	2014	2015	2016	2017	2018	2019	2020
Feeding events	25	48	59	87	102	128	190	173	168	142	159
Total quantity in kg	2,675	12,905	15,670	22,265	30,120	46,845	61,325	56,710	59,370	55,180	56,420

**Table 9.** Total number of Griffon Vultures (*Gypsfulvus*) observed at the supplementary feeding site of VBNP by months for the period 2010–2020.

Year	Month											
	**Jan**	**Feb**	**March**	**April**	**May**	**June**	**July**	**Aug**	**Sept**	**Oct**	**Nov**	**Dec**
**2010**								2	6	13	8	7
**2011**	6	7	8	8	9	9	9	10	12	12	11	13
**2012**	9	6	9	8	10	11	12	13	13	14	12	11
**2013**	12	12	7	24	21	28	26	23	29	19	20	19
**2014**	23	20	21	26	30	50	44	44	35	45	40	40
**2015**	40	40	40	37	43	40	50	48	53	47	43	46
**2016**	43	44	41	36	45	47	41	43	47	46	37	41
**2017**	37	27	37	25	44	41	43	45	40	41	40	39
**2018**	31	37	31	30	45	45	49	48	42	41	40	42
**2019**	25	29	29	41	62	52	54	61	31	36	28	21
**2020**	20	35	24	48	51	49	50	54	45	41	38	43
**Average**:	25	26	25	31	36	37	38	36	32	32	29	29
